# Transmission Pathways of Foot-and-Mouth Disease Virus in the United Kingdom in 2007

**DOI:** 10.1371/journal.ppat.1000050

**Published:** 2008-04-18

**Authors:** Eleanor M. Cottam, Jemma Wadsworth, Andrew E. Shaw, Rebecca J. Rowlands, Lynnette Goatley, Sushila Maan, Narender S. Maan, Peter P. C. Mertens, Katja Ebert, Yanmin Li, Eoin D. Ryan, Nicholas Juleff, Nigel P. Ferris, John W. Wilesmith, Daniel T. Haydon, Donald P. King, David J. Paton, Nick J. Knowles

**Affiliations:** 1 Institute for Animal Health, Pirbright Laboratory, Pirbright, Woking, Surrey, United Kingdom; 2 Division of Environmental and Evolutionary Biology, University of Glasgow, Glasgow, United Kingdom; 3 Animal Health and Welfare, Defra, London, United Kingdom; University of California San Francisco, United States of America

## Abstract

Foot-and-mouth disease (FMD) virus causes an acute vesicular disease of domesticated and wild ruminants and pigs. Identifying sources of FMD outbreaks is often confounded by incomplete epidemiological evidence and the numerous routes by which virus can spread (movements of infected animals or their products, contaminated persons, objects, and aerosols). Here, we show that the outbreaks of FMD in the United Kingdom in August 2007 were caused by a derivative of FMDV O_1_ BFS 1860, a virus strain handled at two FMD laboratories located on a single site at Pirbright in Surrey. Genetic analysis of complete viral genomes generated in real-time reveals a probable chain of transmission events, predicting undisclosed infected premises, and connecting the second cluster of outbreaks in September to those in August. Complete genome sequence analysis of FMD viruses conducted in real-time have identified the initial and intermediate sources of these outbreaks and demonstrate the value of such techniques in providing information useful to contemporary disease control programmes.

## Author Summary

Foot-and-mouth disease (FMD) outbreaks in the United Kingdom during August and September 2007 have caused severe disruption to the farming sector and cost hundreds of millions of pounds. Investigating and determining the source of these outbreaks is imperative for their effective management and future prevention. Foot-and-mouth disease virus (FMDV) has a high mutation rate, resulting in rapid evolution. We show how complete genome sequences (acquired within 24–48 h of sample receipt) can be used to track FMDV movement from farm to farm in real time. This helped to determine the most likely source of the outbreak, assisted ongoing epidemiological investigations as to whether these field cases were linked to single or multiple releases from the source, and predicted the existence of undetected intermediate infected premises.

## Introduction

Foot-and-mouth disease (FMD) is an economically devastating vesicular disease of domesticated and wild cloven-hoofed animals. FMD is caused by a 30 nm un-enveloped virus belonging to the genus *Aphthovirus* in the family *Picornaviridae.* Its genome consists of a single strand of positive-sense RNA approximately 8.3 kb in length [1] encoding a single polyprotein which is post-translationally processed by virally-encoded proteinases [2]. FMD viruses (FMDV) are divided into seven immunologically distinct serotypes known as O, A, C, South African Territories (SAT) 1, SAT 2, SAT 3 and Asia 1. FMDV has a high mutation rate resulting in rapid evolution and extensive variation between and within serotypes [3].

The molecular epidemiology of FMDV has been extensively studied [4],[5]; and has allowed the tracing of outbreak origins on a global scale [4]. Most of these studies have been conducted using nucleotide sequences of one of the three major capsid-coding genes (VP1) which represents less than 10% of the genome. However, VP1 sequence data alone does not have the required resolution for within-epidemic transmission tracing. In common with some other RNA viruses, for example, human immunodeficiency virus (HIV) [6], hepatitis C virus (HCV) [7] and SARS coronavirus [8], full genome sequence for FMDV has recently been used for high-resolution molecular epidemiological studies [9]. To date, fine scale tracing of pathogen transmission has focussed on retrospective analysis; production of full-genome sequences during the course of an outbreak (in real-time) may assist in the interpretation of field epidemiology data and directly influence measures to control the spread of the disease.

**Figure 1:**
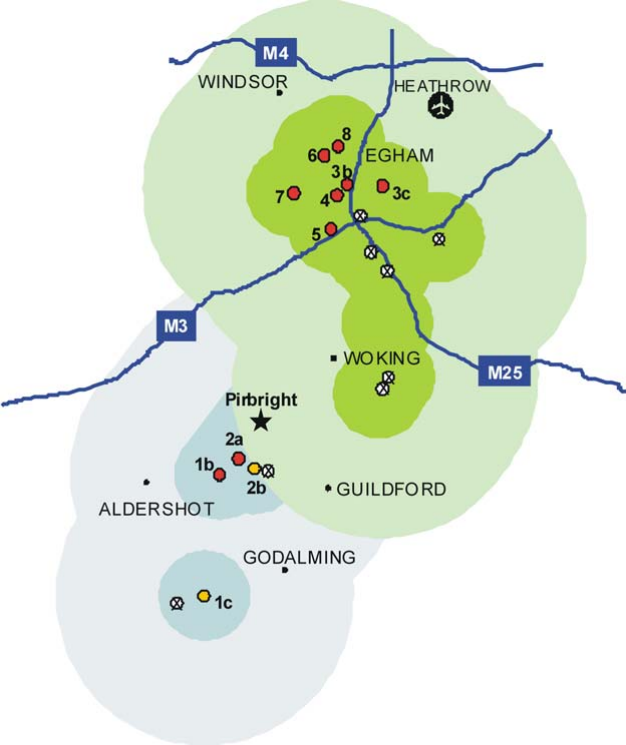
The geographical area affected by FMD outbreaks in 2007. The location of premises and holdings are shown (red circles, clinical signs confirmed by laboratory analysis; yellow circles, FMDV detected using laboratory assays in the absence of clinical disease; and ⊗, additional holdings associated with FMD infected premises with no evidence of infection). The shaded areas denote the extent of the 5km protection zones and 10 km surveillance zones established (blue and green representing outbreaks in August and September respectively). The map also shows major towns and motorways in the region and the location of the Pirbright site (star).

The UK 2007 FMD outbreaks have been characterised by the emergence of two temporally and spatially distinct clusters. Eight infected premises (IP1-8: designation of IP numbering is according to The Department for Environment, Food and Rural Affairs [Defra], UK) have been identified (Figure 1 and Table 1), two in August and six in September. The first case (IP1b) was recognised in beef cattle in a field off Westwood Lane, Normandy, Surrey, UK. Samples collected on 3^rd^ August 2007 from animals exhibiting suspect clinical signs were submitted to the World Reference Laboratory for FMD located at the Institute for Animal Health (IAH), Pirbright, Surrey. Within 24 hours, FMDV sequence data obtained from the first IP (holding IP1b) revealed a VP1 gene-identity of 99.84% to FMDV O_1_ British Field Sample 1860 (O_1_ BFS 1860); intratypic identities between type O VP1 sequences may be as low as 80% [4]. O_1_BFS 1860 is a widely used reference and vaccine strain, originally derived from bovine tongue epithelium received at the World Reference Laboratory for FMD at Pirbright in 1967 from a farm near Wrexham, England. The Pirbright site, comprising the laboratories of the IAH and Merial Animal Health Limited (Merial), is situated 4.4 km from the first IP. Both laboratories were working with the O_1_ BFS 1860 virus strain, making this site a likely source of the outbreak. Three days after the case at IP1b, a second infected premises (IP2b) was identified at Willey Green, approximately 1.5 Km from IP1b. Cattle at a further holding (IP2c) near to and under the same ownership as IP2b were found to be incubating disease at the time of slaughter. Animals on both the affected farms were destroyed and the premises were disinfected. Subsequent clinical and serological surveillance within a 10 km control zone found no evidence of further dissemination of FMD. However, on 12^th^ September 2007, five weeks after the IP1 and IP2 cattle had been culled, FMD was confirmed on the holding of a new IP (IP3b) situated outside the 10 km control zone surrounding IP1 and IP2 (Figure 1). FMD outbreaks were subsequently reported on an additional holding (IP3c) and five more premises (IP4, 5, 6, 7 and 8) all located close to IP3b and outside the original surveillance area (Figure 1).

**Table 1:** Details of FMDV infected premises and the virus isolates that were sequenced.

IP/holding designation	Premises	Date sample collected	Date Sequence analysis reported to Defra	WRLFMD Ref. No.	GenBank accession no.	Date animals examined	Species sampled	Sample	Estimated maximum lesion age on the animal sampled (days)	Oldest lesion on farm (days)	Affected/total stock
IP1b	Westwood Lane, Normandy, Surrey.	03/08/2007	10/08/2007	UKG/7/2007	EU448371	04/08/2007	bovine	epithelium	6	10	38/38 cattle
		04/08/2007	14/08/2007	UKG/7B/2007	EU448372		bovine	epithelium	3		
IP2b	Willey Green, Aldershot Road, Guildford, Surrey.	06/08/2007	23/08/2007	UKG/93/2007	EU448373	07/08/2007	bovine	epithelium	6	7	44/49 cattle
IP2c	Frog Grove Lane, Wood Street Village, Guildford, Surrey.	07/08/2007	29/08/2007	UKG/150/2007	EU448374	07/08/2007	bovine	blood	none	none	0/58 cattle
IP3b	Stroude Road, Egham, Surrey.	12/09/2007	13/09/2007	UKG/643/2007	EU448375	12/09/2007	bovine	epithelium	4–5	5	36/47 cattle
IP3c	Chertsey Lane, Staines, Surrey.	15/09/2007	16/09/2007	UKG/1153/2007	EU448376	15/09/2007	bovine	epithelium	not known	5	9/15 cattle
IP4b	Whitehall Lane, Egham, Surrey.	13/09/2007	15/09/2007	UKG/800/2007	EU448377	13/09/2007	bovine	epithelium	5–7	10	54/54 cattle
IP5	Bridge Lane, Virginia Water, Surrey.	17/09/2007	20/09/2007	UKG/1421/2007	EU448378	17/09/2007	ovine	oesophageal/pharyngeal scrapings	not known	21*	10/16 sheep; 16/24 cattle†; 0/2 pigs
IP6b	Windor Road, Old Windsor, Surrey.	21/09/2007	23/09/2007	UKG/1484/2007	EU448379	21/09/2007	bovine	epithelium	2–4	4	2/32 cattle
IP7	Wick Road, Englefield Green, Egham, Surrey.	24/09/2007	26/09/2007	UKG/1679/2007	EU448380	24/09/2007	bovine	epithelium	1–2	5	14/16 cattle
IP8	Magna Carta Lane, Wraysbury, Staines, Berkshire.	29/09/2007	01/10/2007	UKG/2366/2007	EU448381	29/09/2007	bovine	epithelium	2	2	1/54

*: , approximate age due to the difficultly of aging healed lesions.

**†:** , no samples from cattle were available for analysis.

These outbreaks of FMDV in the UK during August and September 2007 have caused severe disruption to the farming sector and cost more than one hundred million pounds. Investigating and determining the source of these outbreaks has been imperative for their effective management and is vital for future prevention. The aim of this study was to trace FMDV movement from farm-to-farm by comparing complete genome sequences acquired during the course of the epidemic. These “real-time” analyses helped to determine the most likely source of the outbreak, assisted ongoing epidemiological investigations as to whether these field cases were linked to single or multiple releases from the source, and predicted the existence of undetected intermediate infected premises that were subsequently identified.

## Results/Discussion

**Figure 2:**
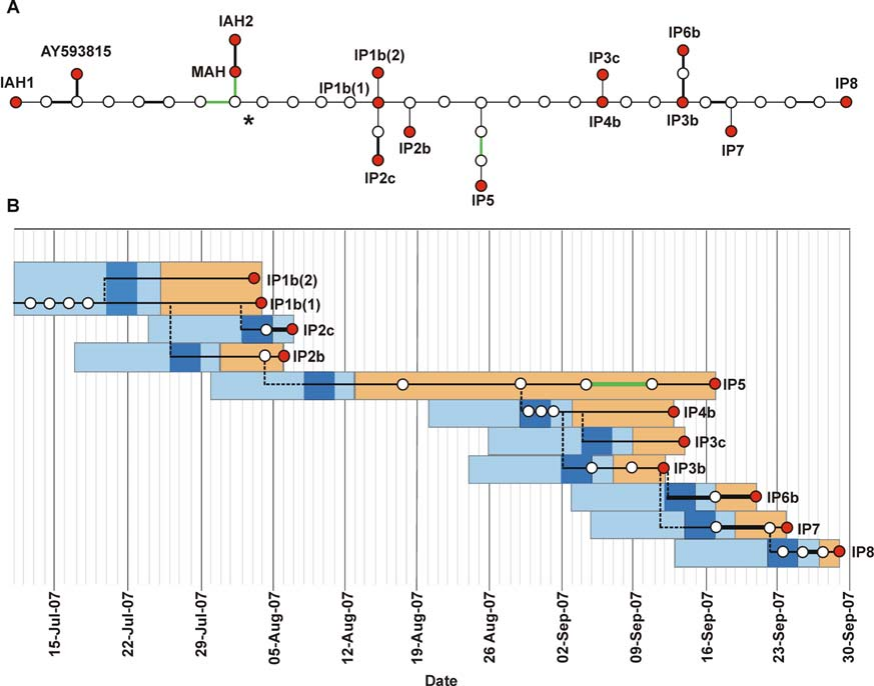
Analysis of sequence data. A) Statistical parsimony analysis by TCS [19] of complete genome sequences of 14 FMDVs; connecting lines represent a nucleotide substitution, thicker lines represent non-synonymous substitutions, with substitutions indicative of adaptation to cell culture coloured green. Sequenced haplotypes (red circles), and putative ancestral virus haplotypes (white circles) are shown. AY593815 is a previously published sequence [21] of FMDV O_1_ BFS 1860. The asterisk indicates the start of the tree in 2B). B) Lesion age derived infection profiles of holdings overlaid with the outbreak virus geneology. The orange shading estimates the time when animals with lesions were present from the oldest lesion age at post-mortem [22]. For IP2c, there were no clinical signs of disease. The light blue shading represents incubation periods for each holding, estimated to begin no more than 14 days prior to appearance of lesions [23]. The dark blue shading is the infection date based on the most likely incubation time for this strain of 2–5 days [24]. Each UK 2007 outbreak virus haplotype is plotted according to the time the sample was taken from the affected animal (x axis). The dashed lines link the TCS tree together but do not denote any genetic change.

The UK 2007 FMD outbreaks were characterised by the emergence of two temporally and spatially distinct clusters. The genetic relationships of FMDV present in eleven field samples from the 2007 outbreak, three cell culture derived laboratory viruses (see Table S1) used at the Pirbright site during July 2007 (designated IAH1, IAH2 and MAH) and a published sequence of O_1_ BFS 1860 (AY593815) are illustrated in Figure 2A. Whereas IAH1 and the virus from which the published sequence was derived are believed to have been passaged no more than ten times in cell cultures, the IAH2 and MAH viruses had been extensively adapted to grow in a baby hamster kidney cell line (Table S1). In natural hosts, FMDV attaches to integrin receptors on the cell surface [10]. However, when grown in cell cultures, the virus may adapt to attach to heparan sulphate (HS), through acquisition of positively charged amino acid residues on the virus coat at positions VP2^134^ and/or VP3^56^ [11],[12]. An additional change from a negatively charged amino acid residue at VP3^60^ to a neutral residue often occurs but may not be essential for HS binding [11]. IAH1 and the previously sequenced isolate of O_1_ BFS 1860 have lysine at VP2^134^, histidine at VP3^56^ and aspartic acid at VP3^60^, none of the residues associated with HS binding, whereas substitutions at VP3^56^ (arginine) and VP3^60^ (glycine) are present in MAH and IAH2, consistent with their history of extensive culture passage (Table 2). The presence of the HS binding-associated substitution at residue VP3^60^ (aspartic acid to glycine) in all but one of the field viruses provides evidence that a cell culture adapted virus is an ancestor of the outbreak. Since this residue is not critical for HS binding it is less likely to undergo reversion [11],[12]. The wild type configurations at VP3^56^ in all of the outbreak viruses and at VP3^60^ in the IP5 virus most likely reflect reversions that have been selected upon replication within the animal host. It is known that there is a strong selection pressure for the reversion at VP3^56^ when FMDV replicates in cattle [11].

**Table 2:** Nucleotide and amino acid substitutions observed in the genomes of the FMD viruses studied.

Position*	AY593815	IAH1	IAH2	MAH	UKG/7B/2007 (IP1b)	UKG/7/2007 (IP1b)	UKG/93/2007 (IP2b)	UIKG/150/2007 (IP2c)	UKG/643/2007 (IP3b)	UKG/1153/2007 (IP3c)	UKG/800/2007 (IP4b)	UKG/1421/2007 (IP5)	UKG/1484/2007 (IP6)	UKG/1679/2007 (IP7)	UKG/2366/2007 (IP8)	Gene	Codon no. in gene†	aa/codon in AY593815	Substituted aa/codon
159	C	C	C	C	C	C	C	C	C	C	C	C	C	C	A	5′ UTR	-	-	-
170	C	C	C	C	T	T	T	T	T	T	T	T	T	T	T	5′ UTR	-	-	-
262	T	T	T	T	T	T	T	T	T	T	T	T	T	C	T	5′ UTR	-	-	-
792	G	G	G	G	G	G	G	A	G	G	G	G	G	G	G	5′ UTR	-	-	-
913	A	A	A	A	A	A	A	A	T	T	T	A	T	T	T	5′ UTR	-	-	-
1083	C	C	A	A	A	A	A	A	A	A	A	A	A	A	A	5′ UTR	-	-	-
1181	G	G	A	G	G	G	G	G	G	G	G	G	G	G	G	Lb	2	E (gaa)	K (aaa)
1951	C	C	C	C	C	T	C	C	C	C	C	C	C	C	C	VP4	-	-	-
2184	G	A	G	G	G	G	G	G	G	G	G	G	G	G	G	VP2	78	C (tgc)	Y (tac)
2377	T	T	T	T	C	C	C	C	C	C	C	C	C	C	C	VP2	-	-	-
2446	C	C	C	C	C	C	C	C	T	T	T	T	T	T	T	VP2	-	-	-
2558	A	A	A	A	A	A	G	A	A	A	A	A	A	A	A	VP2	203	I (att)	V (gtt)
2772	A	A	G	G	A	A	A	A	A	A	A	A	A	A	A	VP3	56	H (cac)	R (cgc)
2784	A	A	G	G	G	G	G	G	G	G	G	A	G	G	G	VP3	60	D (gac)	G (ggc)
3043	A	A	A	A	A	A	A	G	A	A	A	A	A	A	A	VP3	-	-	-
3157	C	C	C	C	C	C	C	C	C	T	C	C	C	C	C	VP3	-	-	-
3433	T	T	T	T	C	C	C	C	C	C	C	C	C	C	C	VP1	-	-	-
3862	C	C	C	C	C	C	C	C	C	C	C	C	C	C	T	VP1	-	-	-
3994	G	G	G	G	G	G	G	G	G	G	G	G	G	A	A	2B	-	-	-
4045	T	T	T	T	T	T	Y	T	T	T	T	T	T	T	T	2B	-	-	-
4407	A	A	A	A	A	A	A	A	A	A	A	A	A	A	G	2B	152	E (gag)	G (ggg)
4639	A	A	A	A	A	A	A	A	G	A	A	A	G	G	G	2C	-	-	-
4927	C	C	C	C	C	C	C	C	C	C	C	T	C	C	C	2C	-	-	-
5152	C	C	C	C	C	C	C	C	C	C	C	Y	C	C	C	2C	-	-	-
5335	C	C	C	C	T	T	T	T	T	T	T	T	T	T	T	2C	-	-	-
5462	G	G	G	G	G	G	G	G	G	G	G	G	G	A	A	3A	32	D (gac)	N (aac)
5473	A	A	A	A	A	A	A	A	G	G	G	G	G	G	G	3A	-	-	-
5492	A	A	C	C	C	C	C	C	C	C	C	C	C	C	C	3A	42	I (atc)	L (ctc)
5592	C	T	T	T	T	T	T	T	T	T	T	T	T	T	T	3A	75	T (acg)	M (atg)
5599	T	C	T	T	T	T	T	T	T	T	T	T	T	T	T	3A	-	-	-
5655	A	A	A	A	A	A	A	A	A	A	A	A	G	A	A	3A	96	K (aaa)	R (aga)
5808	A	A	A	A	A	A	A	A	A	A	A	A	G	A	A	3A	147	E (gag)	G (ggg)
6175	T	T	T	T	T	T	T	T	C	T	T	T	C	C	C	3C	-	-	-
6616	A	A	A	A	A	A	A	A	A	A	A	A	A	A	G	3C	-	-	-
6673	T	T	T	T	T	T	T	T	C	C	C	T	C	C	C	3C	-	-	-
7021	C	C	T	T	T	T	T	T	T	T	T	T	T	T	T	3D	-	-	-
7162	C	C	C	C	C	C	C	C	T	C	C	C	T	T	T	3D	-	-	-
7729	C	C	C	C	C	C	C	C	T	T	T	C	T	T	T	3D	-	-	-
7750	T	T	T	T	T	T	T	T	C	C	C	T	C	C	C	3D	-	-	-
8149	A	A	G	G	G	G	G	G	G	G	G	G	G	G	G	3′ UTR	-	-	-
8152	C	C	C	C	T	T	T	T	T	T	T	T	T	T	T	3′ UTR	-	-	-
8176	A	A	A	A	A	A	A	A	A	A	A	G	A	A	A	3′ UTR	-	-	-
8180	A	A	A	A	A	A	T	A	T	T	T	T	T	T	T	poly(A)	-	-	-

*: , numbered according to AY593815; the following positions were not sequenced: 1–17and 363–466

**†:** , only listed where an amino acid substitution has occurred.

The viruses from the outbreaks differ by at least five unique synonymous substitutions from the laboratory viruses examined (Table 2, Figure 2A). In terms of nucleotide substitutions, two very closely related laboratory viruses (MAH and IAH2) are closest to the sequence of the virus from IP1b (6 and 7 substitutions, respectively) compared with IAH1 (12 substitutions). Viruses IAH2 and MAH differ by only one non-synonymous change at amino acid residue 2 of the Leader-b (Lb) polypeptide (a papain-like cysteine proteinase) (Table 2). Since FMDV is known to exist as variant populations of genetically related viruses [3], it is possible that virus containing the MAH consensus sequence was present as a minority component within the virus population of IAH2. It is also possible that a reversion of the amino acid change at residue 2 of Lb could be selected when the virus goes back into the natural host. Consequently, either of these viruses could be the source of the 2007 outbreak.

Sequence analysis of virus from the first affected holding identified in the second cluster of outbreaks (IP3b) demonstrated that it had evolved from virus from the first cluster of outbreaks (Figure 2A and B). The sequence data are not consistent with a second escape of virus from the Pirbright site, as the virus from IP3b shares five common nucleotide changes with IP1b and IP2c and six in common with IP2b. A Bayesian majority rule consensus tree, Figure S1, estimated in MrBayes [13] indicated that the group linking the second cluster of outbreaks to the first is strongly supported with a posterior probability greater than 0.999. An alternative explanation that these outbreaks arose as a result of a second release of virus that contained this combination of mutations already is difficult to quantify precisely, however, calculations using the highest estimate of population heterogeneity (determined from *in-vitro* experiments; [14]) indicate that this probability is still many magnitudes less likely than a single release (data not shown).

During the second phase of the epidemic, analysis of the data (within 24–48 hours: see Table 1) were rapidly reported to Defra to inform field investigations. As an example, the virus from IP3b was nine nucleotides different from the virus from IP1b (Table 2, Figure 2A). This is a high number of changes for a single farm-to-farm transmission (a retrospective study of virus genomes acquired from sequentially infected farms during the UK 2001 outbreak in Darlington, County Durham, found a mean of 4.5 (SD 2.1) nucleotide changes [15]), and we predicted that there were likely to be intermediate undetected infected premises between the first outbreaks in August and IP3b. Subsequent field investigations discovered IP4b and IP3c, which differed by one nucleotide from each other. IP4b was three nucleotides closer to virus from the first outbreaks, and IP3c also branched off the tree at this point. However, there were still six nucleotide differences between FMDV sourced from IP4b and FMDV sourced from the August outbreaks. Serosurveillance of all sheep within 3 km of the September outbreaks revealed another infected premises (IP5), on which it was estimated that disease had been present for at least two, and possibly up to five weeks. As Figure 2B shows, IP5 is a likely link between the August and September outbreaks.

Epidemiological investigations suggest that animal movements were not involved in the transmission of virus between premises, but a variety of local spread mechanisms (such as movements of contaminated persons, objects and aerosols) could account for the transmission within each geographic and temporal cluster. Although the epidemiological link between the August and September clusters is not known, the genetic data provide strong evidence to link FMDV transmission between these and the other infected farms. The consensus sequences from individual farms were found to differ by 1–5 nucleotide substitutions. It is probable that the variation in number of changes observed (between premises) have resulted from a number of factors including variation in the degree of bottleneck on the transmitted virus population by different transmission routes and number of virus replication cycles that have occurred in the host post-transmission. The genetic relationships between viruses from individual animals shown in Figure 2A and B follows an identical topology to the Bayesian majority rule consensus tree (Figure S1) and in-group relationships are strongly supported by posterior probabilities on genome groupings that were never less than 99%. Although a more confident resolution of the IP-to-IP transmission pathways might be achieved by characterising additional virus haplotypes present on individual holdings, previous sequencing of virus from different animals from the same farm conducted following the UK 2001 outbreaks indicated very limited intra-farm sequence variability [9]. Furthermore, the relationships presented here reveal a transmission pathway between outbreaks that is consistent with the estimates of when holdings became infected and infectious (Figure 2B). The small number of nucleotide substitutions observed between viruses from source and recipient IP suggests that there has been direct transmission without the involvement of other susceptible species, e.g. sheep or deer.

## Materials and Methods

### Genome amplification and sequencing

Total RNA was extracted directly from a 10% epithelial suspension using the RNeasy Mini Kit (Qiagen, Crawley, West Sussex), or from blood or oesophageal/pharyngeal scrapings using TRIzol (Invitrogen, Paisley, UK). Reverse transcription of the RNA was performed using Superscript III reverse transcriptase (Invitrogen) and an oligo-dT primer (see Table S2). Twenty four PCR reactions per genome were performed with Platinum Taq Hi-Fidelity (Invitrogen), using 23 primer sets tagged with forward and reverse M13 universal primer sequences, and one primer set with a oligo-dT reverse primer to obtain the very 3′ end genomic sequence (Table S2). The PCR products overlap such that each nucleotide is covered by two products. The reactions were run on a thermal cycling programme of 94°C for 2 min, followed by 40 cycles of 94°C for 30 s, 55°C for 30 s, 72°C for 1 min, with final step at 72°C for 7 min. Sequencing reactions were performed using the Beckman DTCS kit, with M13 universal forward and reverse primers and specific forward and reverse primers for each PCR product. This resulted in an average of 7.4 times coverage of each base.

### Sequence analysis

The raw data was assembled using the Lasergene® 7 Software package (DNASTAR, Madison, WI) and all further sequence manipulations were performed using BioEdit (version 7.0.1 [16]) and DNAsp (version 3.52 [17]). The data were analysed by statistical parsimony methods [18] incorporated in the TCS freeware [19]. A Bayesian majority rule consensus tree (based on 10,000 trees sampled from 10 million generations) was estimated in MrBayes [13] assuming a General Time Reversible model of nucleotide substitution with invariant sites (the model most strongly supported by more extensive genome data from the UK 2001 outbreak, [15]. This analysis was performed on consensus sequences as supported by previous analysis of within individual viral diversity of naturally infected animals based on results from cloning the capsid genes (the most variable parts of the genome) that show almost 50% of cloned sequences to be identical to the consensus sequences and with an average pi (*π*) value of 7×10^−4^ [20].

### Accession numbers

The FMDV genome sequences have been submitted to the GenBank/EMBL/DDBJ and assigned the accession numbers EU448368 to EU448381.

## Supporting Information

Table S1 Passage histories of the reference viruses studied.
(0.03 MB DOC)

Table S2 Oligonucleotide primers used for the amplification and sequencing of the FMDV genomes studied.
(0.11 MB PDF)

Figure S1 A Bayesian majority rule consensus tree of all sequences included in this study.
(0.03 MB PDF)
