# Supplementary material for: Transmission Pathways of Foot-and-Mouth Disease Virus in the United Kingdom in 2007
Source: PLoS Pathog. 2008 Apr 18;4(4):e1000050. doi: 10.1371/journal.ppat.1000050 (PMC2277462; doi:10.1371/journal.ppat.1000050)
Supplement: Table S1 — Passage histories of the reference viruses studied. (0.03 MB DOC) [file ppat.1000050.s001.doc]

Table S1. Passage histories of the reference viruses studied.

| **Identifier** | **Passage history** | **Genbank accession no.** | **Origin** |
| --- | --- | --- | --- |
| O_1_ BFS 1860 | Presumed CP1, BTy3, LK1 | AY593815 | Carrillo et al., 2005 |
| O_1_ BFS 1860 (IAH1) | BTy4, BHK2 | EU448368 | In use at the IAH. |
| O_1_ BFS 1860 (IAH2) | Between 18 and 24 passages in BHK cells including at least one passage in suspension cells. | EU448369 | In use at the IAH; derived from the Wellcome vaccine strain. |
| O_1_ BFS 1860 (MAH) | Unknown | EU448370 | Vaccine seed virus used by Merial Animal Health Ltd. |

CP, cattle passage

BTy, primary bovine thyroid cells

BHK, BHK-21 (baby hamster kidney) cells

LK, lamb kidney cells.
