# Supplementary material for: Transmission Pathways of Foot-and-Mouth Disease Virus in the United Kingdom in 2007
Source: PLoS Pathog. 2008 Apr 18;4(4):e1000050. doi: 10.1371/journal.ppat.1000050 (PMC2277462; doi:10.1371/journal.ppat.1000050)
Supplement: Table S2 — Oligonucleotide primers used for the amplification and sequencing of the FMDV genomes studied. (0.11 MB PDF) [file ppat.1000050.s002.pdf]

Table S2. Oligonucleotide primers used for the amplification and sequencing of the FMDV genomes studied.

PCR set	Primer name*	Primer sequence (5' to 3')†	Location on genome‡	Amplicon size (bp)§
1	BFS-1F	TTGAAAGGGGGCRCTAG	1-17	413
	BFS-379R	GGGGGGGGGGGGTGAAA	363-379	
2	BFS-370F	CCCCCCCCCCCCCTAAG	370-386	735
	BFS-1070R	AGGTGCCGGCCTCCGGT	1054-1070	
3	BFS-720F	TTTGACTCCACGCTCGA	720-736	735
	BFS-1420R	GTGCTTGATGTTCCAGATC	1402-1420	
4	BFS-1068F	CCTTTCCTTTTATAACCACTGA	1068-1089	735
	BFS-1768R	GTTGTTTATTATGCTGCCAG	1749-1768	
5	BFS-1418F	CACTTGCTCCACACCGGC	1418-1435	735
	BFS-2118R	ACAACTCTGGTCTCGAGA	2101-2118	
6	BFS-1773F	ACATGCAGCAGTATCAAAAC	1773-1792	735
	BFS-2473R	GTGAACCTTGTACTGGTCG	2455-2473	
7	BFS-2123F	GCAGAACGGTTTTTCAAAAC	2123-2142	735
	BFS-2823R	ACCCTGTCCGAGTCTGTT	2806-2823	
8	BFS-2476F	GCCTTGGACCCTTGTGGTT	2476-2494	735
	BFS-3176R	GTGTAATTTGAAACAAGCAG	3157-3176	
9	BFS-2826F	TTGCTCAGTTTGATATGTCT	2826-2845	735
	BFS-3526R	GGTGAGGTCTCCCTCGTG	3509-3526	
10	BFS-3176F	CATGGCAAGGCCGACGGC	3176-3193	735
	BFS-3876R	TTCTGTTTGTGTCTGGCTTCA	3856-3876	
11	BFS-3526F	CTGGGTTCCAAATGGAGC	3526-3543	741
	BFS-4232R	CGGTGTCGGCCAGCATGAT	4214-4232	
12	BFS-3876F	AAATTGTGGCACCGGTGA	3876-3893	735
	BFS-4576R	CTGCTTTTCAAGGATGCCAG	4557-4576	
13	BFS-4226F	GACACCGGTCTCGAGATT	4226-4243	735
	BFS-4926R	AAGTCCTTGCCGTCAGGGT	4908-4926	
14	BFS-4576F	GCGGGATCTGAACGACC	4576-4592	734
	BFS-5275R	TTGAGGCTTGAACATGTCTTG	5255-5275	
15	BFS-4929F	AATACTTTGCCCAAATGGTCT	4929-4949	732
	BFS-5626R	CACCATTTTCTGCCTCTTG	5608-5626	
16	BFS-5276F	CCACCCCTCCAGAATGTG	5276-5293	725
	BFS-5966R	CCTTCACGACCGGGGCT	5950-5966	
17	BFS-5626F	GGATGATGCAGTGAATGAG	5626-5644	735
	BFS-6326R	CGCGGTTCCCACGGTGGA	6309-6326	
18	BFS-5976F	ACGAGGGACCGGTGAAGA	5976-5993	735
	BFS-6676R	GTGATGTGGTTCGGGGTC	6659-6676	
19	BFS-6328F	GAGGGACATCACGAAGCAC	6328-6346	732
	BFS-7025R	CTGTRTCTGGTTCCATGGC	7007-7025	
20	BFS-6676F	CGAGGGGTTGATTGTGGA	6676-6693	735
	BFS-7376R	ACTGGGCGAAGTGTGTGC	7359-7376	
21	BFS-7026F	CGCCCGGCCTCCCCTGG	7026-7042	742
	BFS-7733R	CCTCGAAGTCCAAGTCATAAT	7713-7733	
22	BFS-7376F	TACAGAAACGTGTGGGATGT	7376-7395	624
	BFS-7965R	CCTGCCACGGAGATCAACTTCT	7944-7965	
23	BFS-7685F	TACGGAGACGACATCGTG	7685-7702	543
	BFS-8193R	TTTTTTTTTTTTTTTGATTAAGG	8172-8193	

24	BFS-7685F	TACGGAGACGACATCGTG	7685-7702	536
	UKFMD/Rev6	GGCGGCCGCTTTTTTTTTTTTTTT	poly(A)	
-	Uni-1F	GTAAAACGACGGCCAGT	-	
-	Uni-1R	CAGGAAACAGCTATGAC	-	

*, the last letter indicates a Forward or Reverse primer.
†, a duplicate set of primers (except for UKFMD/Rev6) were synthesized which were preceeded by M13 forward (Uni-1F) or reverse (Uni-1R) primer sequences.
‡, numbering according to GenBank sequence AY593815.
§, amplicon size includes 34 bp derived from the M13 sequences at the 5′ end of each primer.
