# Supplementary material for: Transmission Pathways of Foot-and-Mouth Disease Virus in the United Kingdom in 2007
Source: PLoS Pathog. 2008 Apr 18;4(4):e1000050. doi: 10.1371/journal.ppat.1000050 (PMC2277462; doi:10.1371/journal.ppat.1000050)
Supplement: Figure S1 — A Bayesian majority rule consensus tree of all sequences included in this study. (0.03 MB PDF) [file ppat.1000050.s003.pdf]

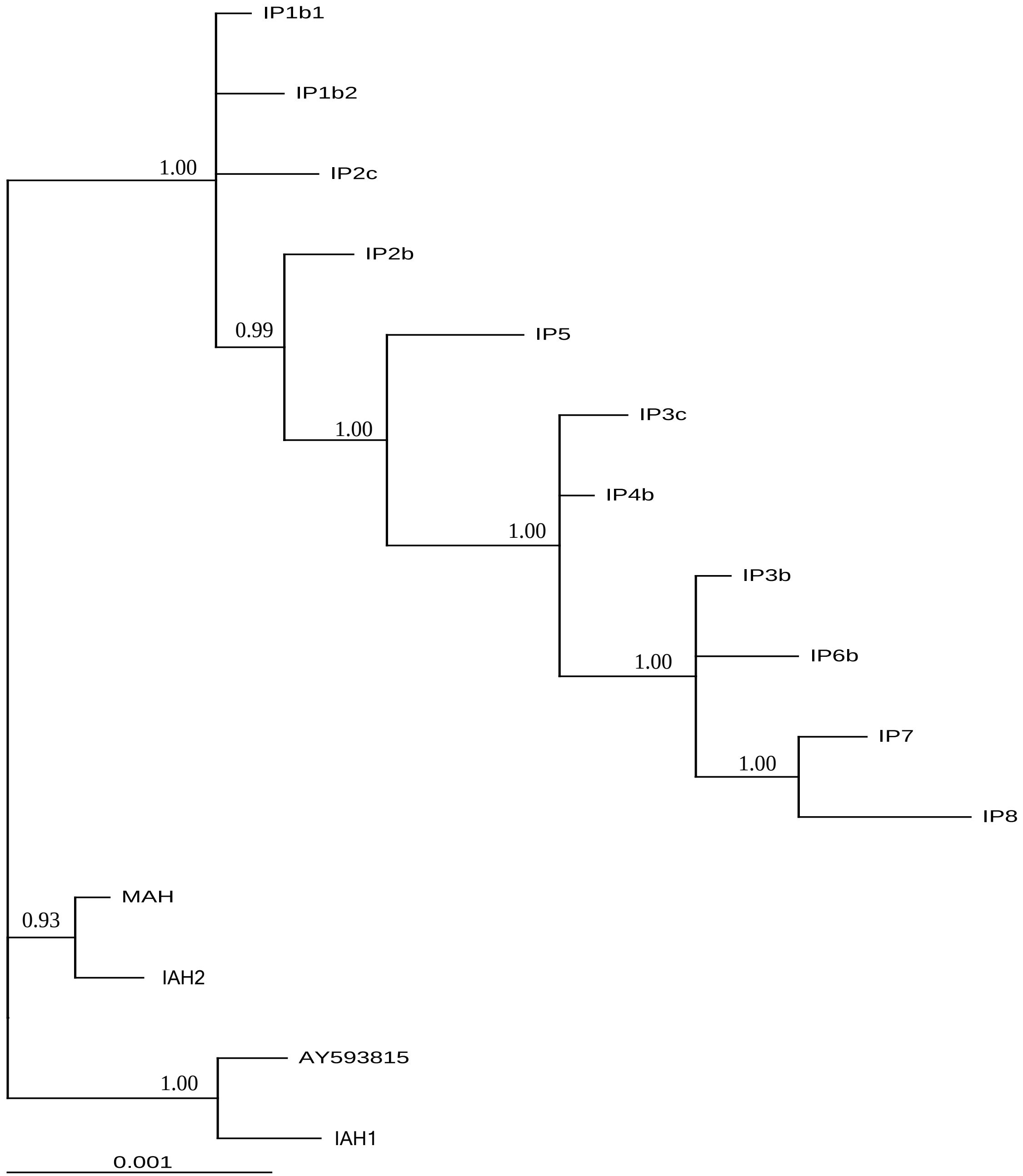

Fig. S1. A Bayesian majority rule consensus tree estimated in MrBayes with posterior probabilities indicated in the branches.
